# Utility of ancillary studies in the diagnosis and risk assessment of Barrett’s esophagus and dysplasia

**DOI:** 10.1038/s41379-022-01056-0

**Published:** 2022-03-08

**Authors:** Won-Tak Choi, Gregory Y. Lauwers, Elizabeth A. Montgomery

**Affiliations:** 1 University of California at San Francisco, Department of Pathology, San Francisco, CA 94143 USA; 2 H. Lee Moffitt Cancer Center and Research Institute, Department of Pathology, Tampa, FL 33612 USA; 3 University of Miami Miller School of Medicine, Department of Pathology and Laboratory Medicine, Miami, FL 33136 USA

**Keywords:** Oesophageal cancer, Barrett oesophagus, Biomarkers

## Abstract

Barrett’s esophagus (BE) is a major risk factor for the development of esophageal adenocarcinoma (EAC). BE patients undergo periodic endoscopic surveillance with biopsies to detect dysplasia and EAC, but this strategy is imperfect owing to sampling error and inconsistencies in the diagnosis and grading of dysplasia, which may result in an inaccurate diagnosis or risk assessment for progression to EAC. The desire for more accurate diagnosis and better risk stratification has prompted the investigation and development of potential biomarkers that might assist pathologists and clinicians in the management of BE patients, allowing more aggressive endoscopic surveillance and treatment options to be targeted to high-risk individuals, while avoiding frequent surveillance or unnecessary interventions in those at lower risk. It is known that progression of BE to dysplasia and EAC is accompanied by a host of genetic alterations, and that exploration of these markers could be potentially useful to diagnose/grade dysplasia and/or to risk stratify BE patients. Several biomarkers have shown promise in identifying early neoplastic transformation and thus may be useful adjuncts to histologic evaluation. This review provides an overview of some of the currently available biomarkers and assays, including p53 immunostaining, Wide Area Transepithelial Sampling with Three-Dimensional Computer-Assisted Analysis (WATS^3D^), TissueCypher, mutational load analysis (BarreGen), fluorescence in situ hybridization, and DNA content abnormalities as detected by DNA flow cytometry.

## Introduction

Barrett’s esophagus (BE) is a pre-neoplastic condition that is associated with an increased risk of developing dysplasia and esophageal adenocarcinoma (EAC)^1–3^. It is defined as endoscopically visible columnar epithelium containing goblet cells (intestinal metaplasia). Although the American Gastroenterological Association has not specified a length requirement^1^, the American College of Gastroenterology requires extension at least 1 cm proximal to the gastroesophageal junction^4^. BE is a genetically unstable metaplastic epithelium that accumulates an increasing number of genetic and chromosomal abnormalities as it progresses to low-grade dysplasia (LGD), high-grade dysplasia (HGD), and eventually EAC^5–10^. Because dysplasia is currently the primary clinical biomarker used to identify patients who are at an increased risk for EAC, clinical guidelines recommend that BE patients undergo periodic endoscopic surveillance with biopsies to detect dysplasia^4^. This allows for risk stratification and management of BE patients based upon the presence and grade of dysplasia prior to the development of EAC. The detection of HGD usually prompts endoscopic therapy, generally in the form of radiofrequency ablation (RFA) with or without endoscopic mucosal resection (EMR), due to its more frequent association with EAC compared with LGD^11–16^. However, a significant number of BE patients with dysplasia (~20%) are resistant to endoscopic therapy, and recurrences or progression to EAC during endoscopic therapy are not uncommon^12,17–23^. As for LGD, although continued surveillance every 12 months is an acceptable approach, there has been a shift toward endoscopic ablation therapy in recent years^4,14–16,24–26^. Subsequently, there is greater emphasis on optimization of the diagnosis of dysplasia as well as identification of patients who are more likely to progress to HGD/EAC and/or have a poor response to endoscopic therapy. However, considering the annual cancer risk for patients with non-dysplastic BE (NDBE) is low (0.1–0.5% per year)^27–29^, identification of reliable biomarkers of low risk to allow prolongation of surveillance intervals compared with the current recommendations of repeating endoscopy every 3–5 years in those with NDBE^4^ remains an important goal of biomarker research.

Even though endoscopic therapy has revolutionized the treatment of BE patients, the current surveillance protocols based on the histologic classification of BE have several shortcomings, including limited sampling of the affected BE segment (leading to false negative biopsy results), sampling error of potentially neoplastic lesions, and interobserver variability among pathologists in the diagnosis and grading of dysplasia, particularly for LGD^30–35^. In fact, there is evidence that the current surveillance protocols may not be effective in reducing mortality from EAC, with one study demonstrating that patients with fatal disease were nearly as likely to have received surveillance (55%) as were controls (60%)^36^. Furthermore, the rate of missed HGD/EAC (i.e., diagnosed within 1 year of negative endoscopy) is high (19–24%)^37^, suggesting that early repeat endoscopy, ideally within 1 year of an initial BE diagnosis, may be crucial, although the cost-effectiveness of this approach remains to be determined. Notably, in a recent meta-analysis of 24 cohort studies of NDBE or LGD patients followed for at least 3 years after index endoscopy, Visrodia et al. reported that ~25% of EACs and 27% of HGD/EACs were classified as missed^37^. When only NDBE patients were considered, the rates of missed EACs and HGD/EACs were 24% and 19%, respectively^37^.

Consequently, there is an increased interest in ancillary tests that could (1) improve the diagnostic accuracy of dysplasia (and its grading) in challenging situations to avoid a repeat endoscopic examination with biopsies (potentially more expensive than most ancillary tests); (2) predict which NDBE or LGD patients are at a higher risk for developing HGD/EAC (including missed lesions) so that such patients can be identified early and successfully treated with endoscopic therapy to prevent progression to EAC; (3) identify patients who are less likely to develop HGD/EAC so that the surveillance of low-risk patients can be reduced; and/or (4) predict those more likely to have a poor response to endoscopic therapy. In this regard, a variety of biomarkers and assays, such as p53 immunostaining^38–42^, Wide Area Transepithelial Sampling with Three-Dimensional Computer-Assisted Analysis (WATS^3D^)^43–50^, TissueCypher^51–58^, mutational load analysis (BarreGen)^59–62^, fluorescence in situ hybridization (FISH)^7,63–67^, and DNA content abnormalities as detected by DNA flow cytometry^68–77^ have been extensively evaluated. Although none of these studies have comprehensively evaluated the potential utility of these biomarkers in reducing mortality from EAC compared with the current surveillance standards, they have demonstrated a potential benefit when used in combination with histologic findings to assist in the diagnosis and/or risk stratification of BE and dysplasia. As such, this review provides an overview of these biomarkers and tests that appear most promising based on the availability of multiple published results and/or on their commercial availability.

## Dysplasia as a biomarker for risk stratification

Currently, dysplasia is the primary clinical biomarker used for risk assessment in the surveillance and management of BE patients. Morphologically, dysplasia is defined as unequivocal neoplastic epithelium that remains confined within the basement membrane of the epithelium from which it developed, and it is classified as (1) negative for dysplasia, (2) indefinite for dysplasia (IND), (3) LGD, or (4) HGD^3,30,31,78^. The rationale for its use as the primary clinical biomarker is based on the premise that EAC in BE patients develops through a sequence of molecular (i.e., loss of *CDKN2A* followed by *TP53* inactivation and aneuploidy) and morphologic changes that begin with intestinal metaplasia and then progress from LGD to HGD, and ultimately to EAC^3,5,68,71,78–82^. This is also supported by multiple outcome studies demonstrating a strong correlation of higher EAC rates with increasing levels of dysplasia. While the annual cancer risk for NDBE patients is low (0.1–0.5% per year)^27–29^, HGD is considered a key premalignant step that is associated with a greater risk of either already having EAC or developing it on follow-up (16–100%)^83–88^. The natural history of LGD is more controversial, with variable progression rates ranging from 0.4 to 13.4% per year^89–91^. It is worth emphasizing that it is not often possible to distinguish true progression from missed lesions in these outcome studies. In other words, a patient with LGD may progress from an unsuspected HGD in the same site or elsewhere in the esophagus. In such a case, HGD/EAC detected on follow-up may represent either true progression or a delayed/missed diagnosis.

Unfortunately, dysplasia has a number of limitations as a biomarker. First, dysplasia is often focal and may not be endoscopically visible, so sampling error is a major issue as most surveillance techniques sample only a minority of the BE segment. Although Reid et al. reported that four-quadrant biopsies taken every 1 cm in the BE segment (also known as the “Seattle protocol”) can consistently detect early cancers arising in HGD^92^, most endoscopists do not adhere to this protocol and take too few biopsies, compounding the problem of sampling error. Second, consistent diagnosis and grading of dysplasia by histology is challenging, as exemplified by a relatively high degree of interobserver variability in the histologic classification of BE among pathologists, particularly toward the lower end of the spectrum^30–32,34,93^. The most pronounced variability is linked to the diagnosis of LGD, with a recent study illustrating sub-optimal interobserver agreement for LGD (kappa = 0.11) even among gastrointestinal (GI) pathologists^32^. In another study, up to 85% of LGD cases were downgraded to NDBE or IND following expert pathology review^91^. Even though an excellent interobserver agreement for HGD among GI pathologists has been reported in earlier studies^30,31^, a more recent study demonstrated that upon review of 485 HGD samples from both academic and private centers by experienced GI pathologists, up to 40% of these cases were reinterpreted as LGD, IND, NDBE, or no BE^93^. Consequently, both the American College of Gastroenterology and the American Gastroenterological Association strongly recommend that all potential dysplasia cases be confirmed by at least one experienced GI pathologist before embarking on a management plan^4,94^. This recommendation is further supported by several studies demonstrating a strong correlation between the number of pathologists who agree with a diagnosis of dysplasia and the rate of neoplastic progression. For instance, Skacel et al. showed that the rate of progression was 80% when three GI pathologists agreed on a diagnosis of LGD, while the rate was 41% when two GI pathologists agreed^95^. Finally, even if the issues stated above could be resolved, there are no observable histologic features in NDBE or LGD on hematoxylin and eosin (H&E) staining that can accurately identify those patients most likely to develop HGD/EAC versus remain stable for years. Indeed, recent studies have suggested that many EACs develop through a more direct, accelerated pathway in which *TP53* mutation is followed by doubling of the whole genome, rapidly resulting in genomic instability, oncogenic amplifications, and EAC, rather than through the stepwise accumulation of tumor suppressor alterations^96,97^. This accelerated pathway to EAC might explain in part why endoscopic surveillance is sometimes unsuccessful in detecting dysplasia before the development of EAC in some BE patients^36^. Overall, these results suggest that additional or alternative biomarker(s) may be useful to better risk stratify BE patients.

## p53 immunostaining as a diagnostic and risk stratification biomarker

**Fig. 1:**
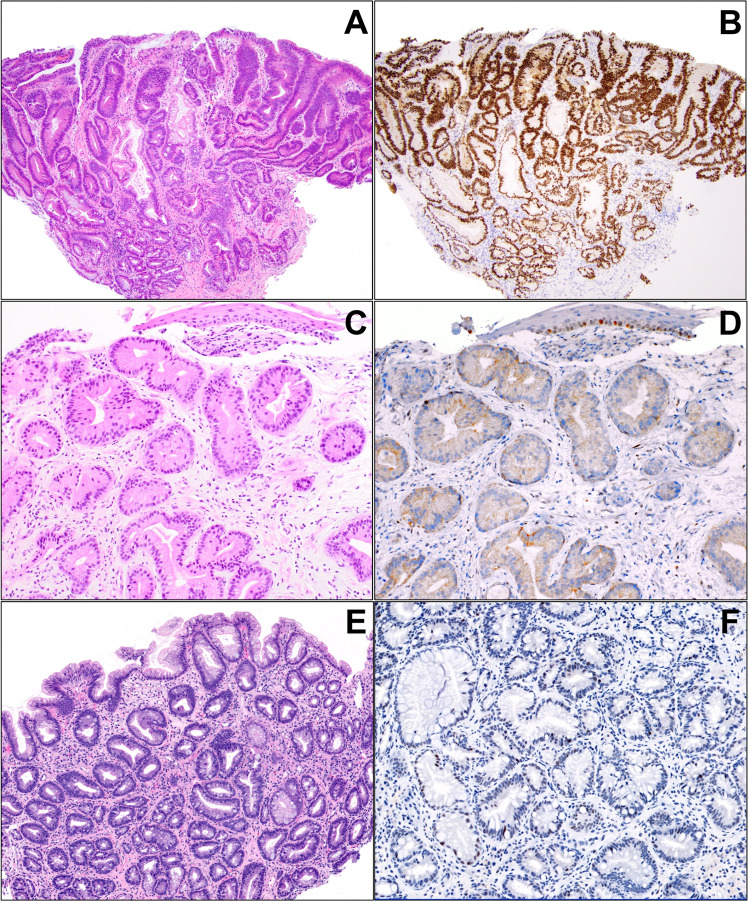
Different p53 expression patterns. **A**, **B** Strong and diffuse p53 overexpression is seen in a case of HGD. **C**, **D** This example shows complete absence of p53 staining (null pattern). The base of the squamous epithelium shows normal positive staining (internal control). **E**, **F** Wild-type pattern of p53 staining in NDBE shows scattered, faintly positive nuclei.

Immunohistochemistry (IHC) for p53 to confirm a dysplasia diagnosis or predict likelihood of progression to EAC is of interest but has limitations, as summarized by others^38,98^. The *TP53* gene encodes p53, which prevents mutations. Normal cells have low levels of this protein in their nuclei, but the gene and protein are upregulated in the presence of DNA damage or stress, resulting in DNA repair, growth attenuation, and apoptosis. In dysplastic cells and EAC, mutations in *TP53* lead to aberrant nuclear accumulation of abnormal p53 protein (which has a long half-life) that can be detected on immunostaining (Fig. 1A, B). Alternatively, truncating mutations/bi-allelic inactivation of *TP53* lead to complete loss of nuclear expression of the protein, termed the “null” pattern (Fig. 1C, D). Light and patchy staining using p53 IHC reflects normal physiologic activity of the protein to maintain cell health and is the pattern of cells that are *TP53* wild-type (Fig. 1E, F). However, in one study of p53 staining, aberrant expression was detected in ~10% of cases regarded as non-dysplastic, ~40% of LGD, ~85% of HGD, and all of EACs^39^. Strong nuclear staining aligns with *TP53* mutations but can still be detected in cases of LGD lacking *TP53* mutations. Bian et al. reported that although 95% of cases interpreted as LGD had p53 expression on IHC, *TP53* mutations were only detected in about a third^40^.

Pathologists in many institutions, particularly in the UK and Europe, have advocated for the use of universal p53 IHC in BE cases to detect dysplasia that might be otherwise overlooked, to the point that the British Society of Gastroenterologists endorsed adding it reflexively in routine practice^99^. The recommendation seemed to reflect studies directed at predicting progression of NDBE to HGD/EAC rather than establishing an initial diagnosis. In one study, scoring p53 immunostaining as “significant” in the presence of strong or absent staining versus “not significant” resulted in kappa scores on the order of 0.6 (strong reproducibility), whereas scoring morphologic features as “negative for dysplasia” versus “IND” versus “LGD” versus “HGD” (4 categories) resulted in kappa scores of 0.3, an unsurprising result since grouping cases into 2 categories versus 4 produces greater observer variability ab initio^41^. In fact, when the authors grouped the morphologic interpretation into only two categories as they had done for p53, namely “definite dysplasia” versus “no dysplasia” on H&E, they achieved a comparable kappa score of 0.55 for morphology alone, diminishing their conclusions concerning p53 considerably. Nonetheless, the results of many studies have supported the use of p53 IHC as a marker of the likelihood of progression to HGD/EAC in patients whose biopsies show H&E findings of negative for dysplasia, IND, or LGD. The latter studies are summarized by Srivastava et al.^38^.

More recently, Redston et al. studied “progressors” versus “non-progressors” gleaned from a large commercial laboratory system^42^. The authors used a retrospective set of over 500 BE patients with or without known progression from negative for dysplasia, IND, or LGD to HGD/EAC. To establish their IHC scoring system (Table 1), the authors obtained DNA for sequencing from 92 BE samples derived from 28 progressors and 6 non-progressors. *TP53* mutations were identified from 50 of the samples, specifically from 21 patients who progressed and 3 who did not. In ~90% of cases, the *TP53* mutational status correlated with p53 immunostaining results. The authors validated their p53 staining criteria using 50 NDBE and 50 HGD biopsies. They found abnormal p53 staining in 4% of NDBE and 96% of HGD, thereby confirming their scoring criteria. In the testing phase, amongst 646 NDBE patients, 20 progressed to LGD, and 10 to HGD/EAC. Abnormal p53 immunostaining was detected in half of the progressors, resulting in good specificity but poor sensitivity. Essentially, amongst 646 NDBE patients, adding p53 staining offered additional information for only 15, and arguably, progression to LGD is not truly progression. The authors suggested that patients with abnormal p53 expression in NDBE have comparable rates of progression to those who have LGD. They further suggested annual endoscopy for such persons. However, the study was limited by the lack of uniformity of the screening and surveillance methods of the gastroenterologists submitting their materials to the commercial laboratory. Also, it is worth noting that although the risk of progression to HGD/EAC is reported to decline with an increased number of endoscopies showing NDBE^100,101^, most studies on ancillary tests, including those of p53, do not clarify the number of negative endoscopies prior to the development of HGD/EAC, complicating the interpretation of their outcome data.

**Table 1:** Scoring method for p53 used by Redston et al.^42^.

p53 expression pattern	Criteria
Normal (wild-type)	2–3+ nuclear staining in ≤20% of epithelial cells in all individual gland bases or glandular profile, and all contiguous stretches of surface epithelium.
Equivocal pattern (subsumed under normal)	2–3+ nuclear staining in 21–50% of epithelial cells in at least one gland base or glandular profile, or within a contiguous focus of at least 20 surface epithelial cells.
Point mutation pattern	2–3+ nuclear staining in >50% of epithelial cells in at least one gland base or glandular profile, or within a contiguous focus of at least 20 surface epithelial cells.
Absent (null) pattern	Total absence of staining in all epithelial cells of at least one gland base or glandular profile. This assumes presence of internal control staining (nuclear positivity of deep glands, base of squamous epithelium, immune cells, or stromal cells).
Cytoplasmic pattern	Total absence of staining in all epithelial cells of at least one gland base or glandular profile. This assumes presence of internal control staining (nuclear positivity of deep glands, base of squamous epithelium, immune cells, or stromal cells).

In a 2018 study, Ten Kate et al. reported that simply refining histologic criteria for diagnosis of LGD identified BE patients likely to progress^102^. Similarly, other refined histologic criteria allowed another group that included one of us (EAM) to essentially eliminate the IND category with excellent prediction of outcome^103^. Ten Kate et al. also used p53 staining alone and achieved similar results to those afforded by use of H&E alone, with some synergy for the two combined but probably not enough to support reflex testing^102^. Years ago, two of us (GYL and EAM) were part of a group that also achieved excellent prediction of outcome using H&E alone despite imperfect interobserver variability^31,104^. We would also point out that the Kaplan–Meier curves for progression of NDBE with and without abnormal p53 staining from the study by Redston et al. do not differ dramatically because so few patients without histologic dysplasia progress regardless of p53 immunostaining status^42^.

Incorporating reflex IHC for p53 is not terribly expensive in the individual patient, and reimbursement is readily obtained. The 2021 Medicare fee schedule listed a 2021 figure of $99.82 for a global code of 88342 (immunostaining; technical only $67.41) and modified it to $106.07 (technical only $70.82), whereas H&E global code (88305) affords $66.76 (technical only $32.06), which was updated to $71.52 (technical only $33.84). This means that adding a p53 stain increases the cost per biopsy by two and a half fold. This might be prohibitively expensive if p53 staining is added to every single esophageal biopsy demonstrating intestinal metaplasia. No cost analysis was provided by Redston et al.^42^, although pathologists might be motivated by payments to add p53 staining to all BE samples that lack dysplasia or show LGD. Overinterpretation of normal staining, however, might result in unnecessary surveillance or ablation procedures.

Most laboratories have used p53 immunostaining for years in evaluation of samples from several organ systems. In esophageal biopsies, however, in expert hands, p53 staining is not necessary to diagnose HGD in BE, which is itself an excellent marker for high risk for progression to EAC^104^, and many gastroenterologists request second opinions for diagnoses of LGD and HGD since either is currently an indication for ablation of the affected segment^4,99^. The updated 2021 Medicare reimbursement fee for the code for outside consultation (88321) is $102.49, which is slightly cheaper than a p53 stain. Adding p53 may have some value in assessing LGD or adding diagnostic precision for cases regarded as IND^103^. However, using positive p53 immunostaining to justify endoscopic therapy in IND or LGD patients, when the concordance between IHC and mutation analysis is less than perfect (~90%), may mean overtreatment in ~10% of patients.

## WATS^3D^ as a diagnostic biomarker

**Fig. 2:**
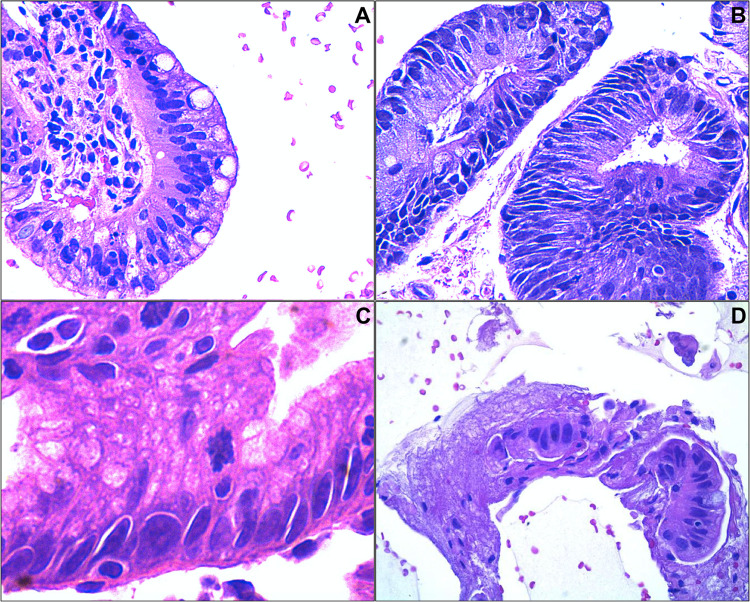
Representative images of WATS^3D^. The images show  (**A**) NDBE, (**B**) LGD, (**C**) HGD, and (**D**) EAC. The images were reproduced with permission from Elsevier^47^.

WATS^3D^ or Wide Area Transepithelial Sampling with Three-Dimensional Computer-Assisted Analysis (CDx Diagnostics, Suffern, NY) is an adjunct test to targeted and random four-quadrant esophageal biopsies using three-dimensional computer-assisted tissue analysis. As discussed previously, the current screening and surveillance guidelines for BE and associated dysplasia require sampling of any visible mucosal abnormality followed by systemic random four-quadrant forceps biopsies obtained at 1–2 cm intervals (Seattle protocol). However, this recommended protocol is time-consuming, labor intensive, and subject to sampling error. As such, the rationale for using WATS^3D^ is to overcome these inherent problems associated with extensive blind sampling^43^. In WATS^3D^, abrasive brushes are used to circumferentially sample the esophageal mucosa. The sampling consists of individual cells as well as mucosal strips reported to measure up to 150 μm in thickness. The material is first analyzed by an imaging system using a neural network optimized for evaluation of the esophageal mucosa. The computer system scans, analyzes, and integrates up to fifty 3-μm optical slices. Ultimately, the system builds three-dimensional images of esophageal glands, and flags goblet cells and dysplastic cells that are displayed for confirmation by a pathologist (Fig. 2). The 2019 guidelines of the American Society for Gastrointestinal Endoscopy conditionally endorsed the use of WATS^3D^ based on low quality evidence for screening and surveillance of BE, in addition to Seattle protocol biopsy sampling for patients with known or suspected BE^44^.

Several studies have demonstrated a significant increase in the detection rates of BE and dysplasia when WATS^3D^ was used adjunctively to the combination of both targeted and random four-quadrant biopsies. For instance, in a large multicenter prospective study of 12,899 patients undergoing BE screening and surveillance and evaluated by 58 community endoscopists, WATS^3D^ was reported to detect additional 213 patients with dysplasia (versus 88 cases detected on biopsy alone), increasing the overall detection of dysplasia by 242%^46^. WATS^3D^ also identified 2570 additional BE cases (versus 1684 BE cases by the combination of targeted and random biopsies alone), increasing the rate of BE detection from 13.1 to 33%. However, among the purported 213 “new” dysplasia cases, 128 (60%) were in fact classified as IND rather than as dysplasia by WATS^3D^, significantly reducing the reported increased detection rate of dysplasia. Furthermore, the increased detection rate of BE was based on the diagnosis of intestinal metaplasia, but the possibility that the cardia was sampled could not be excluded, further weakening the validity of the results.

Only a small series involving 160 BE patients tackled the issue of HGD/EAC detection by WATS^3D^  ^47^. In a multicenter, prospective, randomized trial of referred BE patients at 16 medical centers, Vennalaganti et al. reported that the addition of WATS^3D^ to biopsy sampling yielded additional 23 cases of HGD/EAC. Among these 23 patients, 11 were classified by biopsy as NDBE and 12 as LGD/IND. However, the vast majority of these patients (91.3%) had been previously diagnosed with HGD/EAC on prior biopsies, and most (78%) were confirmed to have HGD/EAC on follow-up biopsies. Also, this study was performed in a high-risk BE population at referral centers, and thus it is not representative of community GI practices and of the BE population at large. Nonetheless, this series is important in that it demonstrates an increased detection rate of high-grade lesions by WATS^3D^, and the biopsy diagnoses had been confirmed by GI pathologists.

Long-term outcome studies of dysplasia diagnosed solely on WATS^3D^ are limited. In a study of over 4000 BE patients who had two WATS^3D^ separated by ≥12 months, Shaheen et al. reported that individuals without dysplasia on WATS^3D^ had a very low risk of progression to HGD/EAC (0.08% per patient-year), while those with LGD had a higher rate of 5.79% per patient-year^50^. However, as noted by the authors, no comparison could be made to the progression rate of LGD detected by microscopy alone. Interestingly, the authors also evaluated the category of crypt dysplasia and reported its risk of progression (1.42% per patient-year) higher than those with no dysplasia but lower than those with LGD. The heterogeneous nature of this diagnostic category likely explains the results.

There are limitations with WATS^3D^. First, although a study examining the interobserver agreement among pathologists using WATS^3D^ found substantial agreement for LGD, HGD, and no dysplasia^48^, the diagnostic criteria used in WATS^3D^ have not been independently tested. Also, the data used to construct the algorithm that creates three-dimensional images of esophageal glands and adequacy criteria used for this test are not well delineated. Furthermore, regenerative epithelial changes in deep glands could be easily misinterpreted as HGD on a single plane of analysis as in WATS^3D^, an issue that merits additional evaluation^49^. Another hindrance to the full validation of this technology is that this commercial test is interpreted by a limited group of pathologists. Independent reproduction and evaluation of WATS^3D^ diagnoses in academic settings with expert reviews of all forceps biopsies by specialized GI pathologists (by all means not infallible) would go a long way to address some of these criticisms. Finally, as noted above, whether the progression rate of dysplasia detected by WATS^3D^ differs from that of dysplasia identified by forceps biopsy and microscopy alone remains to be established.

The issue of additional cost of performing WATS^3D^ has not been extensively evaluated. The cost of WATS^3D^ has been reported to be in the range of $700–800 by our endoscopist colleagues, but whether using this commercial test as an adjunct to traditional endoscopic surveillance is cost effective in the long-term management of BE patients remains to be thoroughly evaluated. Also, the potential value of WATS^3D^ in the era of ever improving advanced endoscopic imaging techniques has not been examined. As endoscopists improve their ability to detect ever more subtle lesions previously described as ‘invisible’, it may lessen the need for broad blind sampling, such as WATS^3D^.

## TissueCypher as a diagnostic and risk stratification biomarker

The objective of TissueCypher is to evaluate samples from BE patients diagnosed as negative for dysplasia, IND, or LGD on routine histologic evaluation to identify those patients most likely to progress to HGD/EAC so that intensified screening or ablation can be offered to them. Similarly, the technique is intended to identify patients who are unlikely to progress such that their surveillance can be reduced.

**Fig. 3:**
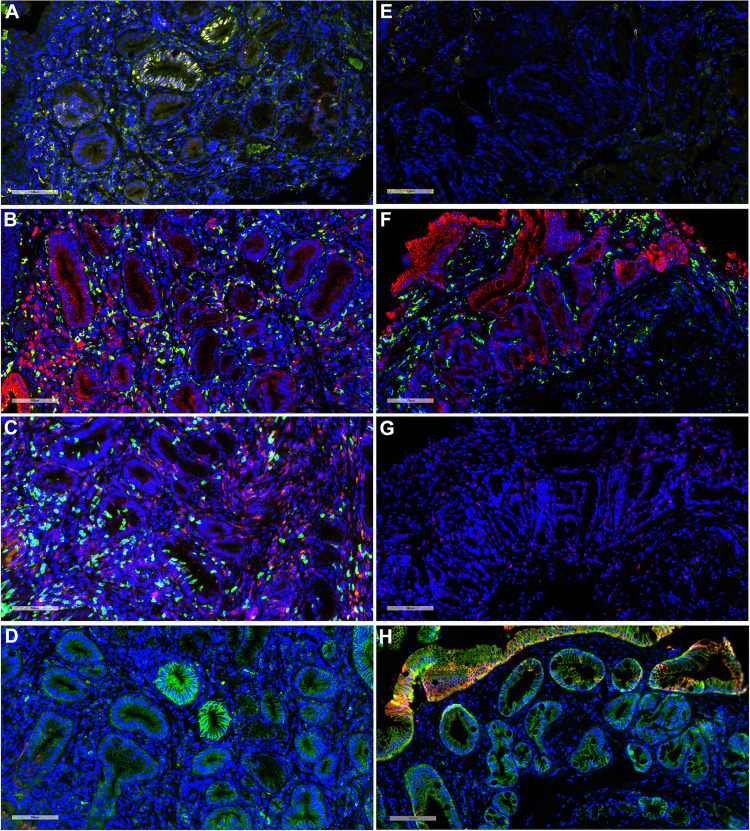
Representative images of TissueCypher. **A–D** show a NDBE biopsy from a 66-year-old man with 8-cm BE segment and who was diagnosed with EAC at a surveillance endoscopy 3.9 years later (progressor). TissueCypher scored this specimen high risk. **E–H** show a NDBE biopsy from a 69-year-old man with 11-cm BE segment with 5.6 years surveillance data showing no progression (non-progressor). TissueCypher scored this specimen low risk. **A** and **E** show p16-green, AMACR-red, and p53-yellow; **B** and **F** show CD68-green and COX-2-red; **C** and **G** show HIF-1α-green and CD45RO-red; **D** and **H** show HER2-green and CK20-red. Nuclei labeled by Hoechst are shown in blue in all panels.

TissueCypher uses immunofluorescent labeling of sections from formalin-fixed paraffin-embedded (FFPE) samples for p16, AMACR, p53, HER2, CK20, CD68, COX-2, HIF-1α, and CD45RO, together with Hoechst staining dye (Fig. 3)^51–58^. Hoechst dye allows fluorescent detection of DNA^105^, thereby permitting image analysis software to identify nuclei as discrete objects in tissue. It also allows the software to assess nuclear area, solidity, and DNA content. Some of the markers are combined on the same slide^51,52^. The slides are then used to perform image analysis with an image analysis algorithm. The image analysis algorithm quantifies 15 different “image features” (Table 2). The quantified image features are then combined into a risk score. Samples are still reviewed in the typical manner (routine diagnosis by local pathologists) and then sections are prepared and subjected to the TissueCypher staining and algorithm.

**Table 2:** Core features assessed by TissueCypher^52^.

Marker	Image analysis feature
p53	p53 nuclear sum intensity
p53	p53 nuclear mean intensity
HER2/neu and CK20	Ratio of mean HER2/neu intensity:mean CK20 intensity in nuclear clusters^b^
HER2/neu and CK20	Ratio of 95^th^ quantile HER2/neu intensity:95^th^ quantile CK20 intensity in nucler clusters
COX-2 and CD68	Co-expression cellular COX-2 mean intensity and cellular CD68 mean intensity
p53	p53 mean intensity in nuclear clusters
p53, p16, and Hoechst^a^/nuclear morphology	Nuclear solidity in p53+ p16– cells
CD45RO	CD45RO plasma membrane sum intensity
AMACR	AMACR microenvironment standard deviation
COX-2	COX-2 texture in cytoplasm^c^
HIF-1α	HIF-1α microenvironment cell mean intensity
HIF-1α	HIF-1α microenvironment cell moment (product of mean and standard deviation)
p16	p16 cytoplasm mean intensity
p53, p16, and Hoechst/nuclear morphology	Nuclear area in p53+ p16– cells
Hoechst/nuclear morphology	Hoechst nuclear 95^th^ quantile intensity

^a^Hoechst is used to stain DNA, which enables image analysis software to segment nuclei as individual objects in tissue images, and to measure nuclear area, solidity, and DNA content.

^b^Nuclear clusters are detected by the image analysis software, and biomarkers are measured within the image regions containing nuclear clusters.

^c^Contrast textural feature is extracted from a co-occurrence matrix and is a measure of the COX-2 intensity contrast between a pixel and its neighbor over the whole tissue image.

This method offers the advantage of using a variety of markers with a consistent interpretation, thus eliminating interobserver variability, although this does not necessarily mean an accurate interpretation. Using the company’s platform, a risk score for progression is stratified as low, intermediate, or high, but there is some advantage to combining the intermediate and high risk scores. Although similar data are reported in all studies from the TissueCypher team^51,52,54–56^, the initial and some recent studies were performed in Europe, and in 2020, a US-based study from two institutions was added^53^. The latter study was a case-control study from patients with biopsy diagnoses of negative for dysplasia (*n* = 227), IND (*n* = 23), and LGD (*n* = 18). The samples were from 58 patients who progressed to HGD/EAC (median time to progression of 2.7 years; 7/58 progressed after 5 years), and from 210 patients who did not progress (median surveillance time of 7 years). In this study, the prevalence-adjusted proportions of patients scoring low, intermediate, and high risk using the TissueCypher method were 84.2%, 9.4%, and 6.4%, respectively. The sensitivity and specificity of the test at 5 years for the 3-tier TissueCypher classification (low, intermediate, and high risk) were 29% and 86%, respectively, and 40% and 86%, respectively, for the 2-tier classification (low and intermediate/high risk combined). By comparison, the sensitivity and specificity of an expert diagnosis of LGD were 19% and 88%, respectively, and the sensitivity and specificity of the initial community diagnoses of LGD (i.e., diagnosis recorded in the health records) were 26% and 66%, respectively. Of 51 patients who progressed within 5 years, 14 scored high risk, 6 scored intermediate risk, and 31 scored low risk. Among 210 patients who did not progress, 13 scored high risk, 18 scored intermediate risk, and 179 scored low risk. Using the TissueCypher test, the prevalence-adjusted positive predictive value (PPV) was 23%; i.e., 23% of patients who score high risk would progress to HGD/EAC within 5 years. The prevalence-adjusted negative predictive value (NPV) was 96.4%. The risk prediction test also showed improved risk stratification when compared to p53 alone using the automated scoring.

Overall, expert pathologists’ diagnosis of LGD outperformed TissueCypher in specificity and PPV, but TissueCypher was more sensitive. However, this was not the case for samples from patients with no dysplasia. Patients without dysplasia as confirmed by expert pathologists who scored high risk were at about 5-fold increased risk of progression as compared to patients without dysplasia who scored low risk using TissueCypher. The adjusted PPV for the test in expert pathologist-confirmed NDBE was 26%, indicating that 26% of patients without dysplasia but with a high risk score using TissueCypher will progress within 5 years, a rate similar to that associated with an expert diagnosis of LGD.

TissueCypher is a send-out test (Cernostics, Pittsburgh, PA) and costs about $5,000. A cost analysis sponsored by the company claimed that it would be cost-effective after 5 years by reducing the number of patients requiring surveillance and reducing EAC-associated deaths^57^. It does not change initial evaluation of patients by routine histology. It has been assigned a CPT code by Medicare (0108U). However, because of the high “up front” costs of TissueCypher, most insurers do not cover the testing. Also, the roughly similar performance characteristics of TissueCypher to histologic evaluation may not justify changing surveillance intervals based on the results, and thus the suggested cost benefit may not materialize, especially given its high cost. As noted above, based on the company’s data, a diagnosis of LGD by an expert pathologist offers more specificity than the test, and obtaining an expert opinion is certainly substantially cheaper. Regardless, the test is consistent, not subject to human observer variation, and outperforms pathologists in identifying patients without dysplasia who are likely to progress to HGD/EAC.

## Mutational load (BarreGEN) as a diagnostic and risk stratification biomarker

Mutational load (ML) analysis provides a measure of cumulative genetic aberrations and instability at 10 key genomic loci by assessing DNA damage around tumor suppressor genes associated with progression to HGD/EAC^59–62^. It can be assessed using a commercially available test (BarreGEN, Interpace Diagnostics, Pittsburgh, PA), with its main objective being to detect dysplasia and risk stratify BE patients. To perform this assay, H&E-stained slides are first evaluated to identify relevant histologic targets (e.g., LGD) that are micro-dissected from 1 to 3 unstained FFPE sections (4 μm in thickness)^60–62,106^. Greater than 90% of each micro-dissected target should contain epithelial cells, from which purified DNA is prepared. Polymerase chain reaction (PCR) and quantitative capillary electrophoresis methods are performed on all micro-dissected areas. ML specifically assesses the presence and extent (clonality) of loss of heterozygosity (LOH) and new alleles consistent with microsatellite instability (MSI) for each micro-dissected target. The following 10 loci are examined, with associated tumor suppressor genes in parentheses: 1p (*CMM1*, *L-myc*), 3p (*VHL*, *HoGG1*), 5q (*MCC*, *APC*), 9p (*CDKN2A*), 10q (*PTEN*, *MXI1*), 17p (*TP53*), 17q (*RNF43*, *NME1*), 18q (*SMAD4*, *DCC*), 21q (*TFF1*, *PSEN2*), and 22q (*NF2*). LOH is categorized as either high (> 75% of the DNA has LOH) or low clonality (50–75% of the DNA has LOH) and assigned values of 1 and 0.5, respectively. The value of the first MSI at a genomic locus is 0.75, and each additional MSI is assigned a value of 0.5. The highest weighted value at each locus is determined based on the values for low and high clonality LOH and MSI at that locus (e.g., the weighted value of high clonality LOH is 1, which is the highest possible weighted value at each of the 10 loci). The sum of all weighted values for all 10 genomic loci is defined as the ML for that micro-dissected target (range: 0–10).

In a case-control study involving 69 BE patients (including 23 progressors and 46 non-progressors), ML assessment was able to risk stratify patients with NDBE or LGD at baseline with respect to progression to HGD/EAC within a mean follow-up time of 4 years^61^. A mean ML score was significantly higher in progressors (ML = 2.2) than non-progressors (ML = 0.4) (*p* < 0.001).^61^ No progressor had a ML of 0 at baseline compared with 54% of non-progressors. Sensitivity was 100% at ML ≥ 0.5, and specificity was 96% at ML ≥ 1.5. Similarly, in a retrospective study of 28 IND patients, patients who progressed to HGD had higher levels of genomic instability (ML ≥ 1.5)^62^. At this threshold, the risk of progression to HGD was 33% (versus 0% in those with an ML < 1.5; *p* = 0.005), with a sensitivity of 100% and a specificity of 85%. Overall, these results indicate that genomic alterations as measured by ML often predate the development of HGD/EAC, potentially allowing ML to be a useful biomarker for predicting disease progression. In addition, Ellsworth et al. demonstrated a signficant correlation of higher ML with increasingly severe histologic grade of BE-associated lesions: ML = 1.1 for IND, 2.2 for LGD, and 3.3 for HGD (*p* < 0.001)^59^. These results suggest that ML may serve as an adjunctive test in patients with equivocal histology.

The biggest appeal of ML assessment is that it allows a direct correlation with morphology and provides an objective quantitative measure of the presence and extent of molecular alterations associated with development of dysplasia and EAC, eliminating human interobserver variability. However, similar to other PCR-based tests, ML assessment may be hampered by insufficient amounts and/or poor quality of DNA as is often the case in FFPE mucosal biopsies. Also, micro-dissection of tiny histologic targets seen on H&E slides is susceptible to sampling error. Another limitation is that purified DNA rather than crude lysate should be used whenever possible, as ML signal in crude lysate could be “muted”^106^. In fact, using crude lysate, there was no difference in mean ML between progressors (ML = 0.73) and non-progressors (ML = 0.74) (*p* = 0.93) in a nested case-control study (involving 48 progressors and 101 non-progressors), failing to validate the previous finding that ML could be useful in risk stratifying BE patients. Finally, as noted above, BarreGEN is not fully validated for commercial use at this time, and it is unclear when this test will be available for clinical use.

## FISH as a diagnostic and risk stratification biomarker

**Fig. 4:**
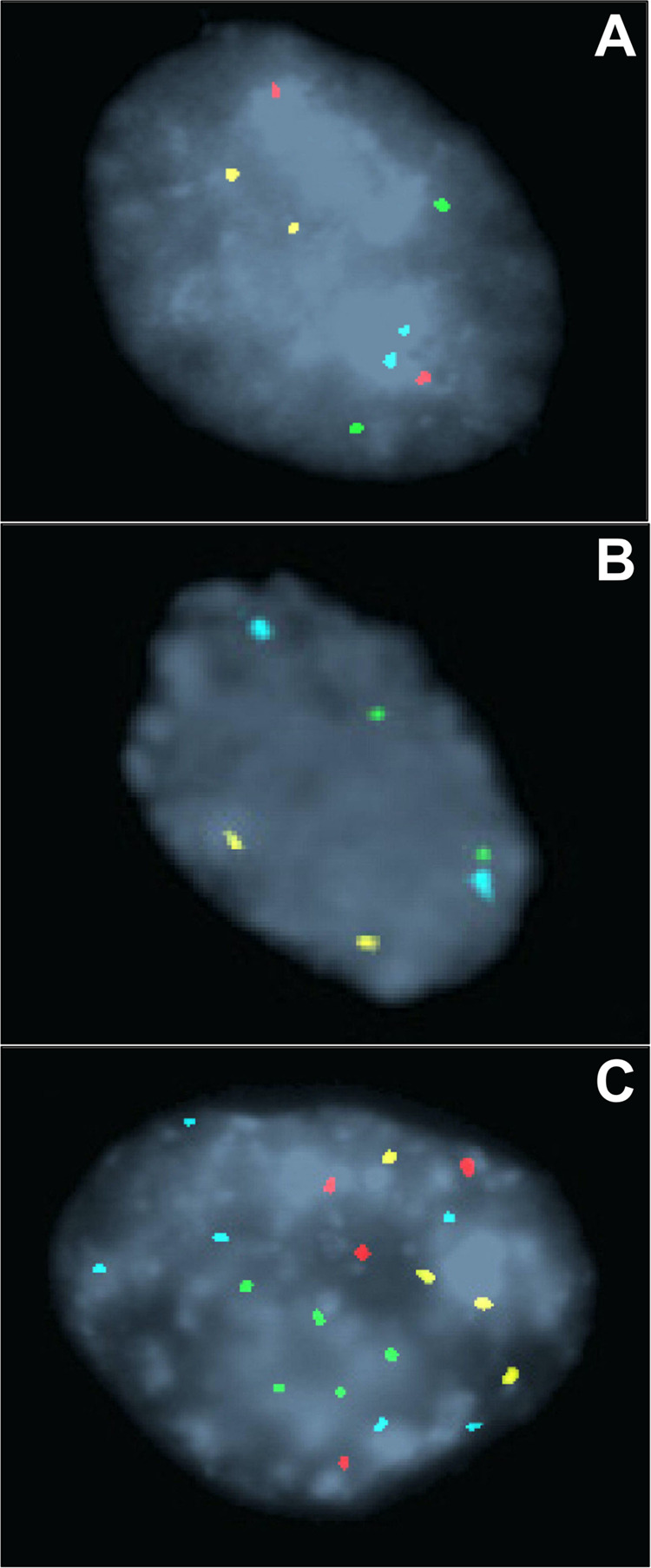
Representative images of FISH signal patterns. **A** Normal FISH result shows 2 signals of each of the 4 probes. **B** Homozygous loss of 9p21 shows no red signal. **C** Polysomic FISH result shows ≥ 3 signals of ≥2 probes. The FISH probes are labeled with Spectrum Aqua (8q24), Red (9p21), Green (17q12), or Gold (20q13) fluorophores. The images were reproduced with permission from Elsevier^63^.

FISH (fluorescent in situ hybridization) is a technique that utilizes fluorescently labeled DNA probes to detect chromosomal abnormalities. To explore its potential utility in the diagnosis and risk stratification of dysplasia in BE patients, several studies, all conducted at Mayo Clinic, utilized a 4 locus-specific probe set targeting 8q24 (*MYC*), 9p21 (*CDKN2A*; alias *P16*), 17q12 (*ERBB2*; alias *Her-2/neu*), and 20q13 (*ZNF217*)^7,63–67^. Each cell is categorized as either normal (i.e., having 2 signals per probe) or abnormal (i.e., having more or less than 2 signals per probe) (Fig. 4). Detectable chromosomal alterations include polysomy (≥3 signals for ≥2 loci), single locus gain (3–9 signals of a single locus and two signals of other loci), amplification of a single locus (≥10 signals of a single locus and two signals of other loci), and single locus loss (0–1 signal of a single locus and two signals of other loci). The test can be performed on either FFPE tissue^7,66^ or endoscopic brushing specimens^63–65,67^.

In a FISH analysis of a range of histologic lesions from 10 esophagectomy specimens from BE patients, polysomy was found to be more prevalent in HGD (88%) and EAC (100%) than in NDBE (<10%) and LGD (57%) (*p* < 0.001), whereas single locus gain was most commonly observed in LGD (28% versus 12% of NDBE versus 8% of HGD)^7^. Also, in a recent multicenter study, if ≥10% of cells had polysomy in the specimen, FISH was able to differentiate between HGD/EAC and the remaining histologic diagnoses with a sensitivity of 80% and a specificity of 88%^66^. Furthermore, in a retrospective analysis of 245 BE patients with a history of HGD, using a cutoff of more than 4 of 100 cells demonstrating polysomy, the risk of EAC was significantly higher within 2 years among patients with a polysomic FISH result (14.2%) compared with those without a polysomic result (1.4%) (*p* < 0.001)^65^. In addition, Timmer et al. demonstrated that in BE patients with HGD or intramucosal adenocarcinoma (IMC)  treated with ablation with or without preceding EMR, polysomy was associated with a lower probability of achieving complete eradication of HGD/IMC (HR = 0.57, *p* = 0.002) in a univariate analysis^67^. Given its high diagnostic accuracy for identifying HGD/EAC and potential to detect HGD/EAC on follow-up, polysomy in combination with histology may be able to serve as a confirmatory marker of HGD/EAC and screening tool to identify patients at highest risk for subsequent detection of HGD/EAC compared with those with non-polysomy.

Despite these promising results, no reference laboratory currently offers this test (apparently due to the lack of demand), although in the past it was available at Mayo Clinic and Neogenomics. However, many academic centers and commercial laboratories routinely run FISH, which can be completed in a few days, and the commercial availability of these probes (~$800 per probe, Abbott Molecular Inc., Des Plaines, IL) allows these laboratories to validate and bring up the same assay if needed. Also, FISH may be more sensitive than other tests such as DNA flow cytometry (described below) by virtue of having the low threshold for a positive polysomy result (e.g., 4 polysomic cells)^63,65,67^. Yet, genetic alterations as detected by FISH are limited to specific gain/loss of genes targeted by the probe set, and thus other non-targeted chromosomal alterations would not be detected, potentially missing some high-risk individuals who could be identified by DNA flow cytometry. Furthermore, FISH often identifies cells with minimal DNA alterations, such as 9p21 (*CDKN2A*) loss, which often do not cause noticeable morphologic abnormalities. Thus, a positive (especially non-polysomy) FISH result does not always indicate the presence of dysplasia.

## DNA content abnormalities as detected by DNA flow cytometry as a diagnostic and risk stratification biomarker

**Fig. 5:**
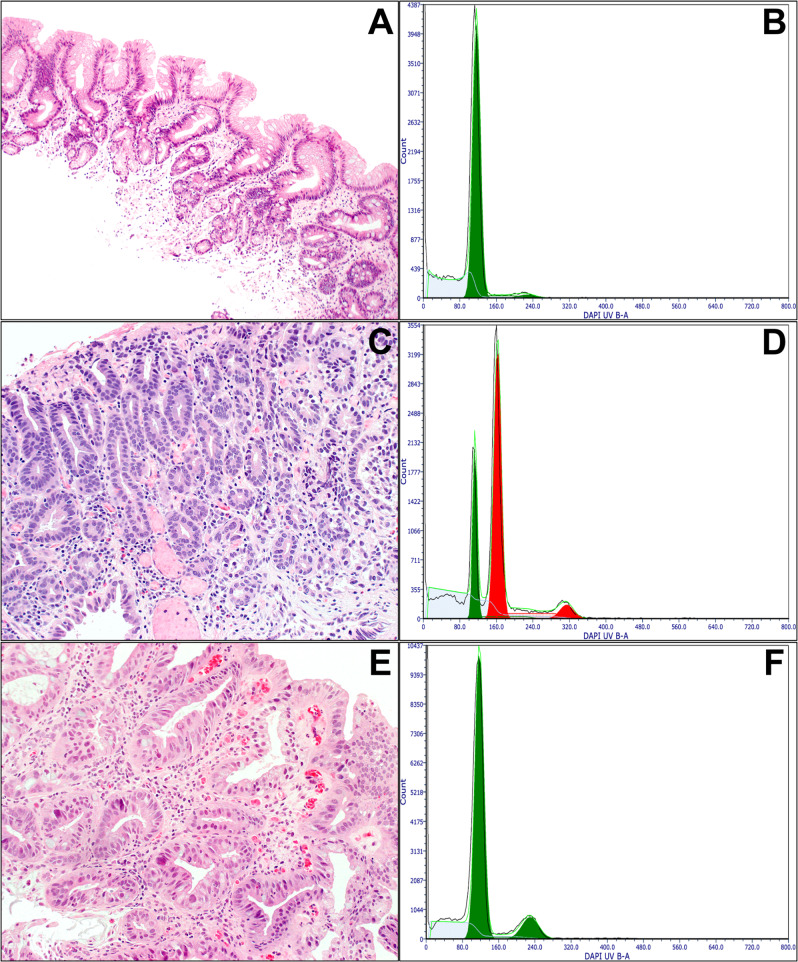
DNA content histograms of NDBE and HGD. **A**, **B** NDBE is characterized by the presence of intestinal metaplasia, but there is no dysplasia. The DNA histogram shows a normal diploid population (green). **C**, **D** HGD is characterized by severe cytologic atypia with enlarged, hyperchromatic, rounder nuclei. The DNA histogram demonstrates a discrete aneuploid peak (red) that is bimodally distinguishable from the normal diploid peak (green). **E**, **F** Another example of HGD shows atypical glands lined by highly pleomorphic cells with enlarged nuclei. There is an elevated 4 N fraction greater than 6% (with DNA index of 1.9–2.1) in the DNA histogram. No distinct aneuploid population is present.

Since the 1980s, a number of studies have consistently demonstrated the potential utility of DNA flow cytometry in the diagnosis and risk stratification of dysplasia in BE patients^68–77^. Although its availability has been limited to few medical centers due to perceived technical demands and use of fresh tissue in earlier studies^68–71^, subsequent studies have successfully employed FFPE tissue for DNA flow cytometric analysis to generate high-quality DNA content histograms, demonstrating the feasibility of this methodology^72–77^. For optimal results, the computer program Multicycle (De Novo software, Glendale, CA) should be used to analyze DNA content histograms^68–71,76,77^. The published consensus guidelines for clinical DNA flow cytometry should be followed^107,108^. Most epithelial cells are normally in the G_0_/G_1_ phase of the cell cycle and have diploid (2 N) DNA content, while less than 6% of cells have tetraploid (4 N) DNA content (G_2_) (Fig. 5A, B). Aneuploidy is defined as an extra G_0_/G_1_ peak that is bimodally separated from the normal diploid G_0_/G_1_ peak (Fig. 5C, D). The presence of a G_2_/tetraploid (4 N) fraction greater than 6% (with DNA index of 1.9–2.1) is also classified as abnormal due to its strong association with neoplasia (Fig. 5E, F)^5,69,71,76,77^.

A recent retrospective study analyzed 80 FFPE BE samples with HGD, 38 LGD, 21 IND, and 14 NDBE and reported that the frequency of DNA content abnormalities (aneuploidy or elevated 4 N fraction) increases with increasing histologic grade of dysplasia: 0% of NDBE, 9.5% of IND, 21.1% of LGD, and 95% of HGD^77^. As a diagnostic marker of HGD, the estimated sensitivity and specificity of abnormal DNA content were 95% and 85%, respectively. Interestingly, DNA flow cytometry also identified a subset of LGD and IND patients who are at higher risk for subsequent detection of HGD/EAC, with the univariate hazard ratios (HRs) of 7.0 and 20.0, respectively (*p* < 0.001)^77^. Considering that endoscopic therapy is increasingly being recommended for LGD patients^26^, abnormal flow cytometric results at baseline LGD or IND could potentially enable clinicians to recommend endoscopic therapy, whereas continued surveillance may be an acceptable approach in the setting of normal flow cytometric results. Furthermore, Bowman et al. recently demonstrated that abnormal DNA content in baseline HGD/IMC can serve as a predictive marker of persistent/recurrent neoplasia following endoscopic therapy, with the univariate and multivariate HRs of 3.8 (*p* = 0.007) and 6.0 (*p* = 0.003), respectively^76^. This suggests that the detection of DNA content abnormalities in baseline HGD/IMC may help to identify high-risk BE patients who may benefit from alternative therapeutic strategies (e.g., different ablation technique, combined endoscopic modalities, or endoscopic submucosal dissection) as well as long-term follow-up with shorter surveillance intervals following endoscopic therapy.

There are some advantages of using DNA flow cytometry. First, DNA flow cytometry is an inexpensive send-out test ($350 at ARUP laboratories; CPT code: 88182) that can be completed within 2–3 days. Second, DNA flow cytometric markers of dysplasia or progression (aneuploidy or elevated 4 N fraction) are usually absent in NDBE^72,75–77,109,110^, and features potentially altering the histologic interpretation (i.e., increased acute inflammation or ulceration) do not cause aneuploidy or elevated 4 N fraction, which can be very helpful in evaluating IND cases^69,111^. In fact, many genetic and chromosomal abnormalities detected in BE (including 9p LOH [site of *CDKN2A*], 17p LOH [site of *TP53*], and mutations of *TP53* and *CDKN2A*) tend to occur early and frequently throughout large areas of BE^5–10,112–114^, even before the first histologic sign of dysplasia, limiting their utility as a diagnostic or prognostic marker of dysplasia in BE patients.

In conclusion, as the current surveillance methods based on the histologic diagnosis and classification of dysplasia imperfectly assess the risk of BE patients, especially those with IND or NDBE histology, there is an increasing demand for ancillary tests to aid in the diagnosis/grading of dysplasia and risk stratification of BE patients. In cases with equivocal histology, one may argue that a repeat endoscopic examination with biopsies may provide the answer without the need of an ancillary test. However, this approach is likely to be more expensive than most ancillary tests. In this regard, several biomarkers and assays, including p53 IHC, WATS^3D^, TissueCypher, mutational load assessment (BarreGen), FISH, and DNA content abnormalities as detected by DNA flow cytometry have been demonstrated as ways to support a dysplasia diagnosis and aid in risk assessment for the development of HGD/EAC (Table 3). More importantly, many of these tests are currently available in academic centers and commercial laboratories, and often utilize FFPE, obviating the need to obtain separate samples. Although none of these tools are widely used in practice, there is an increased interest among gastroenterologists to pursue ancillary tests in BE surveillance biopsies, as they have shown promising results in identifying early neoplasia and could potentially serve as adjuncts to histologic evaluation. By providing information that cannot be assessed by morphology alone, especially if the cost is reasonable (i.e., cheaper than repeat endoscopy with additional pathologic evaluation), these tests may become attractive tools, especially for patients with inconsistent diagnoses, IND, or LGD histology. Like many molecular tests (e.g., next-generation sequencing) currently used in the diagnosis and management of many diseases, incorporating these tools in the management of BE patients, in conjunction with histologic evaluation, may allow for more precise surveillance and/or earlier treatment in patients at higher risk of progression, while avoiding unnecessary interventions or surveillance in those at lower risk. Prospective studies on these biomarkers (including assessment of their potential utility in reducing mortality from EAC) as well as cost-effective analysis compared with the current surveillance methods are singularly missing. Until these comprehensive data exist, it is impossible to fully evaluate their potential impact and better tailor their potential roles in the care of BE patients.

**Table 3:** Summary of biomarkers or ancillary tests.

Biomarker or ancillary test	Potential utility	Specimen	Strength	Limitation	Cost
Dysplasia	Predictive marker	FFPE	Established clinical biomarker	Sampling error; interobserver variability	No additional cost
p53 immunostaining	Diagnostic and predictive marker	FFPE	Widely available; not expensive; reimbursement readily obtained	Variable interpretation of staining results	~$100 per biopsy
WATS^3D^	Diagnostic marker	Brushing specimen	May increase the rate of dysplasia or BE detection	Testing algorithm unknown; not independently validated	Send-out (~$700–800)
TissueCypher	Diagnostic and predictive marker	FFPE	Provides consistent interpretation of a variety of biomarkers, eliminating interobserver variability	High cost; performance characteristics roughly similar to histologic evaluation	Send-out (~$5,000)
BarreGen	Diagnostic and predictive marker	FFPE	Provides quantitative measure of molecular alterations, eliminating interobserver variability	May be hampered by insufficient amounts and/or poor quality of DNA; sampling error; not fully validated for commercial use	Send-out (cost unknown)
FISH	Diagnostic and predictive marker	FFPE or brushing specimen	High sensitivity and specificity as a marker of HGD	Not commercially available	~$800 per probe
DNA flow cytometry	Diagnostic and predictive marker	FFPE or fresh tissue	High sensitivity and specificity as a marker of HGD	Not widely available; sampling error	Send-out (~$350)

## References

[1] Spechler SJ, Sharma P, Souza RF, Inadomi JM, Shaheen NJ, American Gastroenterological Association (2011). American Gastroenterological Association technical review on the management of Barrett’s esophagus. Gastroenterology.

[2] Wang KK, Sampliner RE, Practice Parameters Committee of the American College of Gastroenterology (2008). Updated guidelines 2008 for the diagnosis, surveillance and therapy of Barrett’s esophagus. Am. J. Gastroenterol.

[3] Naini BV, Souza RF, Odze RD (2016). Barrett’s esophagus: a comprehensive and contemporary review for pathologists. Am. J. Surg. Pathol..

[4] Shaheen NJ, Falk GW, Iyer PG, Gerson LB, American College of Gastroenterology (2016). ACG clinical guideline: diagnosis and management of Barrett’s esophagus. Am. J. Gastroenterol.

[5] Galipeau PC (1996). 17p (p53) allelic losses, 4N (G2/tetraploid) populations, and progression to aneuploidy in Barrett’s esophagus. Proc. Natl. Acad. Sci. USA.

[6] Galipeau PC, Prevo LJ, Sanchez CA, Longton GM, Reid BJ (1999). Clonal expansion and loss of heterozygosity at chromosomes 9p and 17p in premalignant esophageal (Barrett’s) tissue. J. Natl. Cancer Inst..

[7] Brankley SM (2012). Fluorescence in situ hybridization mapping of esophagectomy specimens from patients with Barrett’s esophagus with high-grade dysplasia or adenocarcinoma. Hum. Pathol.

[8] Ross-Innes CS (2015). Whole-genome sequencing provides new insights into the clonal architecture of Barrett’s esophagus and esophageal adenocarcinoma. Nat. Genet..

[9] Varghese S (2015). Analysis of dysplasia in patients with Barrett’s esophagus based on expression pattern of 90 genes. Gastroenterology.

[10] Walch AK (2000). Chromosomal imbalances in Barrett’s adenocarcinoma and the metaplasia-dysplasia-carcinoma sequence. Am. J. Pathol.

[11] Pouw RE (2010). Efficacy of radiofrequency ablation combined with endoscopic resection for Barrett’s esophagus with early neoplasia. Clin. Gastroenterol Hepatol.

[12] Haidry RJ (2013). Radiofrequency ablation and endoscopic mucosal resection for dysplastic Barrett’s esophagus and early esophageal adenocarcinoma: outcomes of the UK National Halo RFA Registry. Gastroenterology.

[13] Rouphael C, Anil Kumar M, Sanaka MR, Thota PN (2020). Indications, contraindications and limitations of endoscopic therapy for Barrett’s esophagus and early esophageal adenocarcinoma. Ther. Adv. Gastroenterol.

[14] Sharma P, Shaheen NJ, Katzka D, Bergman J (2020). AGA clinical practice update on endoscopic treatment of Barrett’s esophagus with dysplasia and/or early cancer: expert review. Gastroenterology.

[15] Wani S, Standards of Practice Committee (2018). Endoscopic eradication therapy for patients with Barrett’s esophagus-associated dysplasia and intramucosal cancer. Gastrointest Endosc.

[16] Komanduri S, Muthusamy VR, Wani S (2018). Controversies in endoscopic eradication therapy for Barrett’s esophagus. Gastroenterology.

[17] Shaheen NJ (2009). Radiofrequency ablation in Barrett’s esophagus with dysplasia. N. Engl. J. Med..

[18] Anders M (2014). Long-term recurrence of neoplasia and Barrett’s epithelium after complete endoscopic resection. Gut.

[19] Bulsiewicz WJ (2013). Safety and efficacy of endoscopic mucosal therapy with radiofrequency ablation for patients with neoplastic Barrett’s esophagus. Clin. Gastroenterol Hepatol.

[20] Ell C (2000). Endoscopic mucosal resection of early cancer and high-grade dysplasia in Barrett’s esophagus. Gastroenterology.

[21] Peters FP (2005). Endoscopic treatment of high-grade dysplasia and early stage cancer in Barrett’s esophagus. Gastrointest Endosc..

[22] Fujii-Lau LL (2017). Recurrence of intestinal metaplasia and early neoplasia after endoscopic eradication therapy for Barrett’s esophagus: a systematic review and meta-analysis. Endosc. Int. Open.

[23] Cotton CC (2017). Late recurrence of Barrett’s esophagus after complete eradication of intestinal metaplasia is rare: final report from ablation in intestinal metaplasia containing dysplasia trial. Gastroenterology.

[24] Wani S, Rubenstein JH, Vieth M, Bergman J (2016). Diagnosis and management of low-grade dysplasia in Barrett’s esophagus: clinical practice updates expert review from the clinical guidelines committee of the American Gastroenterological Association. Gastroenterology.

[25] di Pietro M, Fitzgerald RC, Barrett’s BSG, BSG Barrett’s guidelines working group (2018). Revised British Society of Gastroenterology recommendation on the diagnosis and management of Barrett’s oesophagus with low-grade dysplasia. Gut.

[26] Phoa KN (2014). Radiofrequency ablation vs endoscopic surveillance for patients with Barrett esophagus and low-grade dysplasia: a randomized clinical trial. JAMA.

[27] Hvid-Jensen F, Pedersen L, Drewes AM, Sørensen HT, Funch-Jensen P (2011). Incidence of adenocarcinoma among patients with Barrett’s esophagus. N. Engl. J. Med..

[28] Wani S (2011). Patients with nondysplastic Barrett’s esophagus have low risks for developing dysplasia or esophageal adenocarcinoma. Clin. Gastroenterol Hepatol.

[29] Desai TK (2012). The incidence of oesophageal adenocarcinoma in non-dysplastic Barrett’s oesophagus: a meta-analysis. Gut.

[30] Reid BJ (1988). Observer variation in the diagnosis of dysplasia in Barrett’s esophagus. Hum. Pathol.

[31] Montgomery E (2001). Reproducibility of the diagnosis of dysplasia in Barrett esophagus: a reaffirmation. Hum. Pathol.

[32] Vennalaganti P (2016). Discordance Among Pathologists in the United States and Europe in Diagnosis of Low-grade Dysplasia for Patients With Barrett’s Esophagus. Gastroenterology.

[33] Moole H (2016). Progression from low-grade dysplasia to malignancy in patients with Barrett’s esophagus diagnosed by two or more pathologists. World J. Gastroenterol.

[34] Sonwalkar SA (2010). A study of indefinite for dysplasia in Barrett’s oesophagus: reproducibility of diagnosis, clinical outcomes and predicting progression with AMACR (alpha-methylacyl-CoA-racemase). Histopathology.

[35] Kaye PV (2009). Barrett’s dysplasia and the Vienna classification: reproducibility, prediction of progression and impact of consensus reporting and p53 immunohistochemistry. Histopathology.

[36] Corley DA (2013). Impact of endoscopic surveillance on mortality from Barrett’s esophagus-associated esophageal adenocarcinomas. Gastroenterology.

[37] Visrodia K (2016). Magnitude of missed esophageal adenocarcinoma after Barrett’s esophagus diagnosis: a systematic review and meta-analysis. Gastroenterology.

[38] Srivastava A (2017). The use of ancillary stains in the diagnosis of Barrett esophagus and Barrett esophagus-associated dysplasia: recommendations from the Rodger C. Haggitt gastrointestinal pathology society. Am. J. Surg. Pathol.

[39] Kastelein F (2013). Aberrant p53 protein expression is associated with an increased risk of neoplastic progression in patients with Barrett’s oesophagus. Gut.

[40] Bian YS, Osterheld MC, Bosman FT, Benhattar J, Fontolliet C (2001). p53 gene mutation and protein accumulation during neoplastic progression in Barrett’s esophagus. Mod. Pathol.

[41] Kaye PV (2016). Dysplasia in Barrett’s oesophagus: p53 immunostaining is more reproducible than haematoxylin and eosin diagnosis and improves overall reliability, while grading is poorly reproducible. Histopathology.

[42] Redston M (2021). Abnormal TP53 predicts risk of progression in patients with Barrett’s esophagus regardless of a diagnosis of dysplasia. Gastroenterology.

[43] Sharma P (2004). Review article: emerging techniques for screening and surveillance in Barrett’s oesophagus. Aliment Pharm. Ther..

[44] Qumseya B, ASGE Standards of Practice Committee (2019). ASGE guideline on screening and surveillance of Barrett’s esophagus. Gastrointest Endosc.

[45] Gross SA, Smith MS, Kaul V, US Collaborative WATS3D Study Group. (2018). Increased detection of Barrett’s esophagus and esophageal dysplasia with adjunctive use of wide-area transepithelial sample with three-dimensional computer-assisted analysis (WATS). U Eur. Gastroenterol J..

[46] Smith MS (2019). Wide-area transepithelial sampling with computer-assisted 3-dimensional analysis (WATS) markedly improves detection of esophageal dysplasia and Barrett’s esophagus: analysis from a prospective multicenter community-based study. Dis. Esophagus.

[47] Vennalaganti PR (2018). Increased detection of Barrett’s esophagus-associated neoplasia using wide-area trans-epithelial sampling: a multicenter, prospective, randomized trial. Gastrointest Endosc..

[48] Vennalaganti PR (2015). Inter-Observer Agreement among Pathologists Using Wide-Area Transepithelial Sampling With Computer-Assisted Analysis in Patients With Barrett’s Esophagus. Am. J. Gastroenterol..

[49] Canto MI, Montgomery E (2018). Response. Gastrointest Endosc..

[50] Shaheen NJ, Smith MS, Odze RD (2021). Progression of Barrett’s esophagus, crypt dysplasia, and low-grade dysplasia diagnosed by wide-area transepithelial sampling with 3-dimensional computer-assisted analysis: a retrospective analysis. Gastrointest Endosc.

[51] Critchley-Thorne RJ (2016). A tissue systems pathology assay for high-risk Barrett’s esophagus. Cancer Epidemiol. Biomark Prev..

[52] Critchley-Thorne RJ (2017). A tissue systems pathology test detects abnormalities associated with prevalent high-grade dysplasia and esophageal cancer in Barrett’s esophagus. Cancer Epidemiol Biomark Prev..

[53] Davison JM (2020). Independent blinded validation of a tissue systems pathology test to predict progression in patients with Barrett’s esophagus. Am. J. Gastroenterol.

[54] Diehl DL, Khara HS, Akhtar N, Critchley-Thorne RJ (2021). TissueCypher Barrett’s esophagus assay impacts clinical decisions in the management of patients with Barrett’s esophagus. Endosc Int. Open.

[55] Frei NF (2021). Tissue systems pathology test objectively risk stratifies Barrett’s esophagus patients with low-grade dysplasia. Am. J. Gastroenterol.

[56] Frei NF (2020). Independent validation of a tissue systems pathology assay to predict future progression in nondysplastic Barrett’s esophagus: a spatial-temporal analysis. Clin. Transl. Gastroenterol.

[57] Hao J, Critchley-Thorne R, Diehl DL, Snyder SR (2019). A cost-effectiveness analysis of an adenocarcinoma risk prediction multi-biomarker assay for patients with Barrett’s esophagus. Clinicoecon Outcomes Res..

[58] Prichard JW (2015). TissueCypher(™): A systems biology approach to anatomic pathology. J. Pathol. Inf..

[59] Ellsworth E, Jackson SA, Thakkar SJ, Smith DM, Finkelstein S (2012). Correlation of the presence and extent of loss of heterozygosity mutations with histological classifications of Barrett’s esophagus. BMC Gastroenterol.

[60] Khara HS (2014). Assessment of mutational load in biopsy tissue provides additional information about genomic instability to histological classifications of Barrett’s esophagus. J. Gastrointest Cancer.

[61] Eluri S (2015). The presence of genetic mutations at key loci predicts progression to esophageal adenocarcinoma in Barrett’s esophagus. Am. J. Gastroenterol.

[62] Trindade AJ (2019). Mutational load may predict risk of progression in patients with Barrett’s oesophagus and indefinite for dysplasia: a pilot study. BMJ Open Gastroenterol.

[63] Barr Fritcher EG (2008). A comparison of conventional cytology, DNA ploidy analysis, and fluorescence in situ hybridization for the detection of dysplasia and adenocarcinoma in patients with Barrett’s esophagus. Hum Pathol.

[64] Brankley SM (2006). The development of a fluorescence in situ hybridization assay for the detection of dysplasia and adenocarcinoma in Barrett’s esophagus. J. Mol. Diagn..

[65] Brankley SM (2016). Fluorescence in situ hybridization identifies high risk Barrett’s patients likely to develop esophageal adenocarcinoma. Dis. Esophagus.

[66] Poneros JM (2017). A multicenter study of a fluorescence in situ hybridization probe set for diagnosing high-grade dysplasia and adenocarcinoma in Barrett’s Esophagus. Dig. Dis. Sci.

[67] Timmer MR (2014). Prediction of response to endoscopic therapy of Barrett’s dysplasia by using genetic biomarkers. Gastrointest Endosc..

[68] Reid BJ, Levine DS, Longton G, Blount PL, Rabinovitch PS (2000). Predictors of progression to cancer in Barrett’s esophagus: baseline histology and flow cytometry identify low- and high-risk patient subsets. Am. J. Gastroenterol.

[69] Reid BJ, Haggitt RC, Rubin CE, Rabinovitch PS (1987). Barrett’s esophagus. Correlation between flow cytometry and histology in detection of patients at risk for adenocarcinoma. Gastroenterology.

[70] Reid BJ (1992). Flow-cytometric and histological progression to malignancy in Barrett’s esophagus: prospective endoscopic surveillance of a cohort. Gastroenterology.

[71] Rabinovitch PS, Longton G, Blount PL, Levine DS, Reid BJ (2001). Predictors of progression in Barrett’s esophagus III: baseline flow cytometric variables. Am. J. Gastroenterol.

[72] Montgomery EA (1996). Barrett esophagus with dysplasia. Flow cytometric DNA analysis of routine, paraffin-embedded mucosal biopsies. Am. J. Clin. Pathol..

[73] Menke-Pluymers MB, Mulder AH, Hop WC, van Blankenstein M, Tilanus HW (1994). Dysplasia and aneuploidy as markers of malignant degeneration in Barrett’s oesophagus. The Rotterdam Oesophageal Tumour Study Group. Gut.

[74] Huang Q, Yu C, Zhang X, Goyal RK (2008). Comparison of DNA histograms by standard flow cytometry and image cytometry on sections in Barrett’s adenocarcinoma. BMC Clin. Pathol.

[75] Kerkhof M (2008). Aneuploidy and high expression of p53 and Ki67 is associated with neoplastic progression in Barrett esophagus. Cancer Biomark.

[76] Bowman CJ (2021). Persistent or recurrent Barrett’s neoplasia after an endoscopic therapy session is associated with DNA content abnormality and can be detected by DNA flow cytometric analysis of paraffin-embedded tissue. Mod Pathol.

[77] Choi WT (2018). Diagnosis and risk stratification of Barrett’s dysplasia by flow cytometric DNA analysis of paraffin-embedded tissue. Gut.

[78] Goldblum JR (2015). Current issues in Barrett’s esophagus and Barrett’s-related dysplasia. Mod. Pathol.

[79] Wild CP, Hardie LJ (2003). Reflux, Barrett’s oesophagus and adenocarcinoma: burning questions. Nat. Rev. Cancer.

[80] Souza RF, Morales CP, Spechler SJ (2001). Review article: a conceptual approach to understanding the molecular mechanisms of cancer development in Barrett’s oesophagus. Aliment Pharm Ther.

[81] Reid BJ (2001). p53 and neoplastic progression in Barrett’s esophagus. Am. J. Gastroenterol.

[82] Reid BJ (2001). Predictors of progression in Barrett’s esophagus II: baseline 17p (p53) loss of heterozygosity identifies a patient subset at increased risk for neoplastic progression. Am. J. Gastroenterol.

[83] Schnell TG (2001). Long-term nonsurgical management of Barrett’s esophagus with high-grade dysplasia. Gastroenterology.

[84] Skinner DB (1983). Barrett’s esophagus. Comparison of benign and malignant cases. Ann. Surg..

[85] Haggitt RC, Tryzelaar J, Ellis FH, Colcher H (1978). Adenocarcinoma complicating columnar epithelium-lined (Barrett’s) esophagus. Am. J. Clin. Pathol..

[86] Schmidt HG, Riddell RH, Walther B, Skinner DB, Riemann JF (1985). Dysplasia in Barrett’s esophagus. J. Cancer Res. Clin. Oncol..

[87] Zhu W (2009). A histologically defined subset of high-grade dysplasia in Barrett mucosa is predictive of associated carcinoma. Am. J. Clin. Pathol.

[88] Williamson WA (1991). Barrett’s esophagus. Prevalence and incidence of adenocarcinoma. Arch. Intern. Med..

[89] Singh S (2014). Incidence of esophageal adenocarcinoma in Barrett’s esophagus with low-grade dysplasia: a systematic review and meta-analysis. Gastrointest Endosc..

[90] Qumseya BJ (2017). Disease progression in Barrett’s low-grade dysplasia with radiofrequency ablation compared with surveillance: systematic review and meta-analysis. Am. J. Gastroenterol.

[91] Curvers WL (2010). Low-grade dysplasia in Barrett’s esophagus: overdiagnosed and underestimated. Am. J. Gastroenterol.

[92] Reid BJ, Blount PL, Feng Z, Levine DS (2000). Optimizing endoscopic biopsy detection of early cancers in Barrett’s high-grade dysplasia. Am. J. Gastroenterol.

[93] Sangle NA (2015). Overdiagnosis of high-grade dysplasia in Barrett’s esophagus: a multicenter, international study. Mod Pathol.

[94] Spechler SJ, Sharma P, Souza RF, Inadomi JM, Shaheen NJ, American Gastroenterological Association (2011). American Gastroenterological Association medical position statement on the management of Barrett’s esophagus. Gastroenterology.

[95] Skacel M (2000). The diagnosis of low-grade dysplasia in Barrett’s esophagus and its implications for disease progression. Am. J. Gastroenterol.

[96] Stachler MD (2015). Paired exome analysis of Barrett’s esophagus and adenocarcinoma. Nat Genet.

[97] Nones K (2014). Genomic catastrophes frequently arise in esophageal adenocarcinoma and drive tumorigenesis. Nat. Commun.

[98] Panarelli NC, Yantiss RK (2016). Do ancillary studies aid detection and classification of Barrett esophagus?. Am. J. Surg. Pathol.

[99] Fitzgerald RC (2014). British Society of Gastroenterology guidelines on the diagnosis and management of Barrett’s oesophagus. Gut.

[100] Kunzmann AT (2021). Does risk of progression from Barrett’s esophagus to esophageal adenocarcinoma change based on the number of non-dysplastic endoscopies?. Dig. Dis. Sci.

[101] Peters Y (2019). Incidence of Progression of Persistent Nondysplastic Barrett’s Esophagus to Malignancy. Clin. Gastroenterol Hepatol.

[102] Ten Kate F (2018). Improved Progression Prediction in Barrett’s Esophagus With Low-grade Dysplasia Using Specific Histologic Criteria. Am. J. Surg. Pathol.

[103] Waters KM, Salimian KJ, Voltaggio L, Montgomery EA (2018). Refined Criteria for Separating Low-grade Dysplasia and Nondysplastic Barrett Esophagus Reduce Equivocal Diagnoses and Improve Prediction of Patient Outcome: A 10-Year Review. Am. J. Surg. Pathol.

[104] Montgomery E (2001). Dysplasia as a predictive marker for invasive carcinoma in Barrett esophagus: a follow-up study based on 138 cases from a diagnostic variability study. Hum Pathol.

[105] Latt SA, Stetten G (1976). Spectral studies on 33258 Hoechst and related bisbenzimidazole dyes useful for fluorescent detection of deoxyribonucleic acid synthesis. J. Histochem. Cytochem.

[106] Eluri S (2018). Validation of a biomarker panel in Barrett’s esophagus to predict progression to esophageal adenocarcinoma. Dis Esophagus.

[107] Shankey TV (1993). Guidelines for implementation of clinical DNA cytometry. International Society for Analytical Cytology. Cytometry.

[108] Rabinovitch P. S. Practical considerations for DNA content and cell cycle analysis. In: Bauer K. D., Duque R. E., and Shankey T. V., editors. Clinical Flow Cytometry: Principles and Applications. Baltimore, MD: Williams and Wilkins, 1992. p. 117-142.

[109] Sikkema M (2009). Aneuploidy and overexpression of Ki67 and p53 as markers for neoplastic progression in Barrett’s esophagus: a case-control study. Am. J. Gastroenterol.

[110] Giménez A (1998). Flow cytometric DNA analysis and p53 protein expression show a good correlation with histologic findings in patients with Barrett’s esophagus. Cancer.

[111] Lee H, Rabinovitch PS, Mattis AN, Kakar S, Choi WT (2020). DNA flow cytometric analysis of paraffin-embedded tissue for the diagnosis of malignancy in bile duct biopsies. Hum Pathol.

[112] Zeki SS (2013). Clonal selection and persistence in dysplastic Barrett’s esophagus and intramucosal cancers after failed radiofrequency ablation. Am. J. Gastroenterol.

[113] Prasad GA (2008). Utility of biomarkers in prediction of response to ablative therapy in Barrett’s esophagus. Gastroenterology.

[114] Prasad GA (2008). Correlation of histology with biomarker status after photodynamic therapy in Barrett esophagus. Cancer.

